# The state of emergency medical services and acute health facility care in Uganda: findings from a National Cross-Sectional Survey

**DOI:** 10.1186/s12913-020-05508-8

**Published:** 2020-07-09

**Authors:** Albert Ningwa, Kennedy Muni, Frederick Oporia, Joseph Kalanzi, Esther Bayiga Zziwa, Claire Biribawa, Olive Kobusingye

**Affiliations:** 1 Department of Disease Control and Environmental Health, Makerere University School of Public Health, Kampala, Uganda; 2 Department of Epidemiology, University of Washington, Seattle, WA USA; 3 Department of Emergency Medical Services, Ministry of Health, Kampala, Uganda

**Keywords:** Emergency medical services, Pre-hospital care, Ambulance

## Abstract

**Background:**

There is limited information on the state of emergency medical services (EMS) in Uganda. The available evidence is from studies that focused on either assessing EMS capacity and gaps at the national level especially in Kampala or identifying risk factors for specific emergency medical conditions (e.g., injuries). In this study, we sought to provide a snapshot of the state of EMS in Uganda by assessing the pre-hospital and hospital emergency care capacity at both national and sub-national (district) levels.

**Methods:**

We conducted a cross-sectional national survey administering structured questionnaires to EMS providers and policy makers from 38 randomly selected districts across seven of the 14 health regions of Uganda. This resulted in a study sample of 111 health facilities and 52 pre-hospital service providers. We collected data on six pillars of EMS whose frequencies and percentages were calculated and qualitatively compared for different levels of the health care system.

**Results:**

At the time of this study, Uganda did not have any EMS policy or guidelines. In addition, there was no functional toll-free number for emergency response in the country. However, Ministry of Health reported that a taskforce had been set up to lead development of EMS policy, guidelines, and standards including establishment of a toll-free emergency number.

At the sub-national level, ambulances lacked the products and supplies needed to provide pre-hospital care, and mainly functioned as emergency transport vehicles, with no capacity for medical care.

Only 16 (30.8%) of the 52 pre-hospital providers assessed had standard ambulances with required equipment, medicines, and personnel. The rest of the service providers had improvised ambulances that were not equipped to provide pre-hospital care.

Traffic police and bystanders were the first responders to the majority (> 90%) of the emergency cases.

**Conclusion:**

Our findings reveal weaknesses at every level of what should be a critical component in the health care system – one that deals with the ability to treat life-threatening conditions in a time sensitive manner. The Ministry of Health needs to speed up efforts to provide policies and guidelines, and to increase investments for the creation of a functional EMS in Uganda.

## Background

An emergency medical service (EMS) system is defined as one that organizes all aspects of care provided to patients in the pre-hospital or out-of-hospital environment [1]. EMS is critical to the improvement of outcomes in patients with obstetric and medical emergencies and severe injuries, and other serious time sensitive illnesses. Despite this pivotal role, many countries in Africa (e.g., Lesotho, Malawi, and Tanzania) have been slow to develop EMS systems [2]. Because the pre-hospital space is not exclusively the purview of the health sector (i.e., may involve other sectors such as police and fire department whose primary mandate is not health), leadership, policy, and practice specific to EMS have been slow to take root both in Africa and elsewhere in low- and middle-income countries [3]. In addition to pre-hospital care, patient outcomes are greatly impacted by the acute care delivered at the receiving health facility [4]. Patient survival and recovery are dependent on the presence of appropriately trained medical personnel, and the availability of the necessary equipment, medicines, and supplies in the minutes and hours following the arrival of a critically ill patient at a health care facility [5].

With a well-established EMS system (i.e., with functional prehospital care, transportation, and hospital care) as is found in most high income countries, many emergency medical conditions can be resolved in a few hours or days [6]. While a few studies have been done to assess pre-hospital care in Kampala [7–9], to our knowledge, no study has been done to assess the status of EMS and acute health facility care in Uganda at national level. The Ministry of Health (MoH) recognized the need to improve these services, and through this study sought to establish the status of emergency medical services and acute health facility care in the country. The assessment was conducted both at the national and sub-national levels assessing EMS capacity at the pre-hospital and facility levels using the World Health Organization (WHO) Emergency Care Systems Assessment (ECSSA) tool [10].

## Methods

### Sample size and sampling methodology

**Fig. 1:**
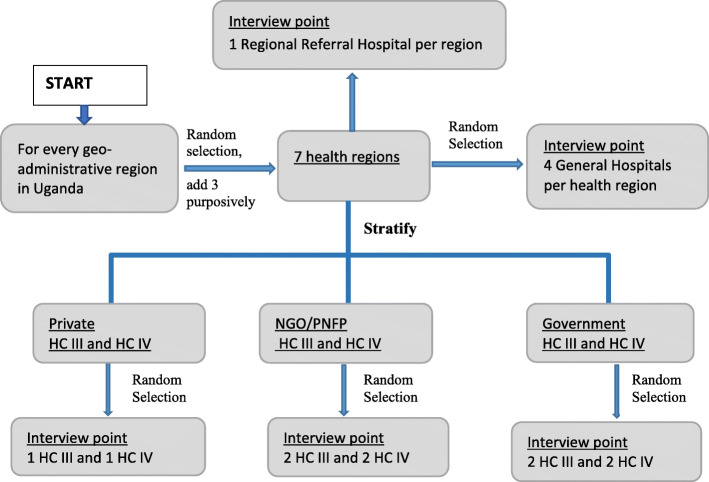
Visual representation of the study sampling methodology

The Uganda health care system is organised into several levels; national referral hospitals, regional referral hospitals, and general (district) hospitals. Within the district, there are health centers with varying capabilities; with Health Center I being the most basic and Health Center IV offering the most comprehensive medical services. Because Health Centers I (individual health volunteer) and II do not care for serious medical conditions [11], they were left out of the sample. We obtained a sampling frame of all health facilities in Uganda from MoH and stratified the list by health regions (there are 14 health regions in the country, with each region served by a regional referral hospital). The health regions were further grouped into Uganda’s 4 geo-administrative regions [12] (i.e., North, East, West, and Central) to ensure each geo-administrative region was represented in the sample. Within each geo-administrative region, we randomly selected one health region (Fig. 1). Within each selected health region, we purposively included the regional referral hospital (RRH) because it is where districts refer cases. We purposively included three additional health regions: Arua health region in West Nile since it hosts a large refugee population, which may impact access and availability of EMS; Karamoja health region since it has a history of conflict (largely from intercommunal cattle raiding) and has historically been disadvantaged with poor access to all social services; and the Kalangala district which is made up of 84 islands and therefore has unique transport access challenges.

Within each selected health region, four general hospitals were randomly selected using computer generated random numbers. In order to select health centers (HCs), we grouped all HCs in the selected health regions by ownership (i.e., government-owned, private not-for-profit/non-governmental organization (PNFP/NGO), and private for-profit HCs). For each health region, we randomly selected 2 private for-profit health centers (i.e., 1 HC IV and 1 HC III), 4 PNFP/NGO health centers (i.e., 2 HC IV and 2 HC III), and 4 government-owned health centers (i.e., 2 HC IV and 2 HC III). Where a private-for-profit or PNFP/NGO HC III or HC IV did not exist in the selected health regions, we filled the slot (s) with a government-owned HC III or HC IV.

Our sampling strategy resulted in a sample size containing 7 regional referral hospitals, 24 general (district) hospitals, 30 HC IV and 30 HC III. In addition, Kampala District was considered a special region due to its status as the capital city with a high concentration of health resources, so out of the three RRHs (i.e., Rubaga, Nsambya, and Naguru) in the city, one RRH (Naguru) was added to the study sample. Additionally, through the guidance of the District Health Officers, 52 pre-hospital care providers (which included the police and ambulance service providers) were identified and included in the study. The police were included as pre-hospital care providers because they are often the first responders at casualty scenes and provide transportation to victims.

**Fig. 2:**
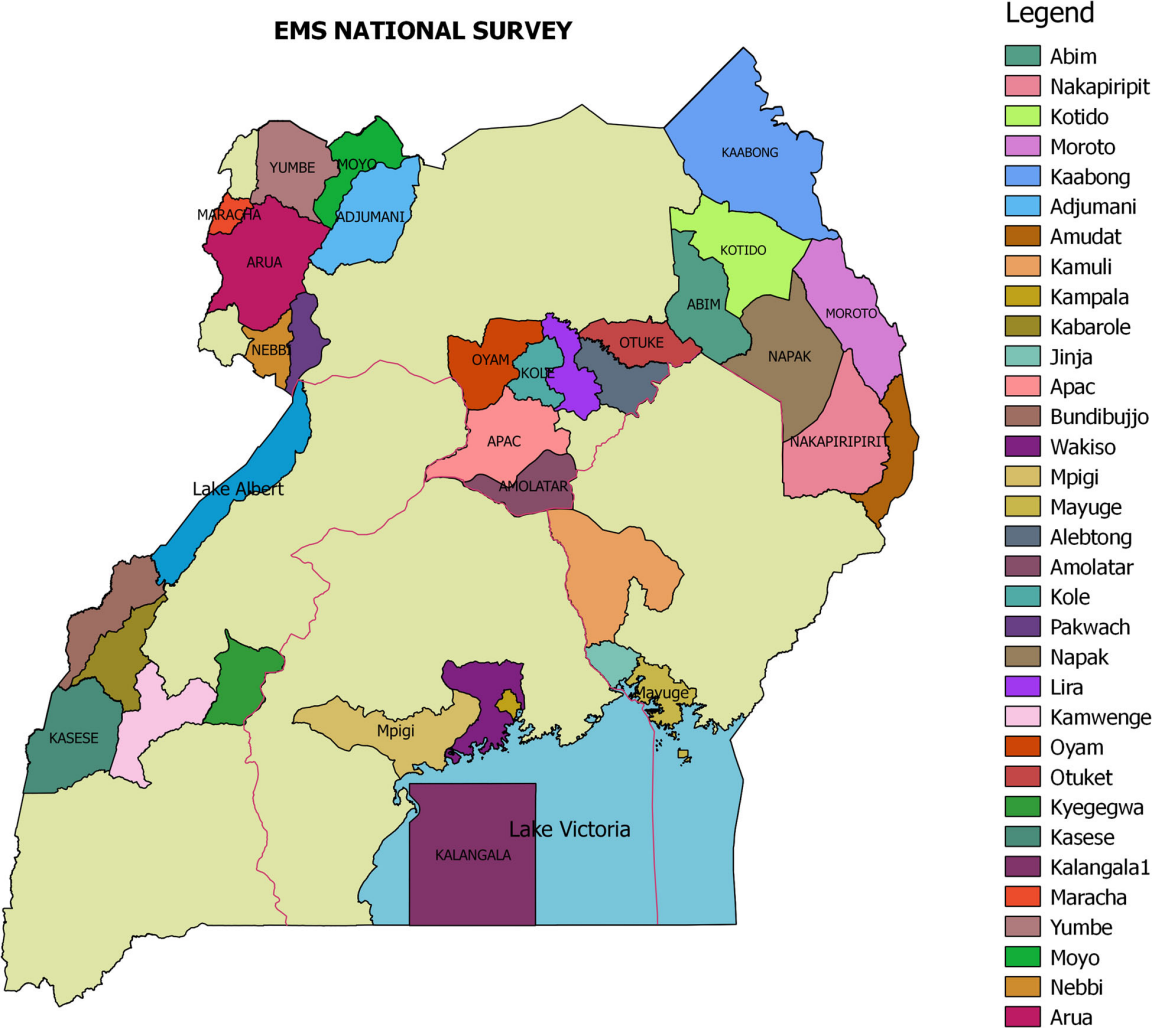
Map of Uganda highlighting districts included in the study. Image Source: Authors [13]

In summary, our study was a cross-sectional national survey whose sample included 7 health regions, 38 districts (Fig. 2) [13], 111 health facilities, and 52 pre-hospital care providers. From each of the 38 districts, we interviewed one senior district officer, most often the District Health Officer who is a district-level decision maker, and a total of 202 key personnel involved in EMS and acute health facility care.

### Data collection

We adapted the WHO Emergency Care Systems assessment tool [14] developed by Teri Reynolds and others [10] to collect data on EMS at the pre-hospital and health facility levels. The tool comprised of checklists and structured questionnaires, which assessed six health system pillars: leadership and governance; financing; information; health workforce; medical products; and service delivery. A pilot of the study was conducted at conveniently and purposively selected hospitals and emergency care providers within Kampala to test the recruitment process, feasibility of the study, and the research tool. As part of the survey, we administered structured questionnaires to the following: district-level policy makers, administrators or managers of pre-hospital care services, and managers of emergency departments or casualty units who we identified through the District Health Officers (DHOs) and hospital administrators. A checklist was used to assess pre-hospital service providers’ premises, ambulances, and emergency units within health facilities through direct observation by teams of trained research assistants, led by medical doctors who tested each equipment to ascertain they were in working condition. We collected data using Open Data Kit (ODK) Collect (version 1.18.2) [15]. Data collection took place between February 12, 2018 and April 24, 2018.

In addition, we reviewed reports from previous EMS studies in Uganda [7–9], and filled gaps in information, especially concerning national-level leadership and governance issues as well as financing, through a key informant face to face interview with a senior MOH official.

### Analysis

We conducted quantitative analyses using STATA 14 (College Station, Texas, USA). The analyses were descriptive (i.e., counts and proportions) and were organized according to the six pillars of the WHO Emergency Care Systems assessment tool [10]. Where applicable, frequencies (counts) and percentages were calculated and qualitatively compared for different levels of the health care system (i.e., referral hospitals, district hospitals and health centers).

We conducted thematic content analysis from the key informant interview with the senior MoH official.

## Results

A summary of the EMS findings both at the national and sub-national levels is presented in Table 1. Detailed discussion of the results by the two levels is presented below.

**Table 1:** A summary of the results highlighting the six EMS pillars

National	Leadership and governance	Strong and committed leadership at MOH. No policy, no guidelines. Development of policy and guidelines underway.
	Financing	No designated funds for EMS.
	Information	Health management information system has no EMS specific data. Data on acute facility care limited.
	Health workforce	Limited staff.
Sub-national	Leadership and governance	No clear lead agency in some districts. No coordination. No policies, guidelines, standards.
	Financing	Low, no ear-marking of funds for EMS. Facility based care funded as part of facility funding.
	Information	Poor generation and use. Limited information sharing.
	Health workforce	Present but limited in EMS. Present in limited numbers, poorly trained in and outside facilities, most facilities have no fixed staff in emergency areas.
	Medical products	Acute shortages in pre-hospital and health facility spaces, even of basics.
	Service delivery	Limited by poor coordination and financing.

### National-level findings

From the interview with the senior MOH official and review of available EMS reports, we found the following at the national level.

#### Leadership and governance

The Department of Emergency Medical Services at the Ministry of Health was a few months old at the time of the study.

‘*The Ministry is prioritizing this [EMS] now, as you can see with the creation of the Department. But the major challenge is still that of resources. We have some development partners who are helping*.’ Key Informant, Ministry of Health.

#### Financing

Specifically, on funding:

‘*There is no earmarking of funds. We get a block vote for salaries and some limited operations here at headquarters. For the rest of the country, the districts have to determine what to spend on it [EMS]. There is no ear marking there*.’ Key informant, Ministry of Health.

#### Coordination

The MOH was acknowledged as the lead agency in this area; however, there was an overlap of roles and ambiguity in mandates and operations, between the MOH, Office of the Prime Minister’s National Emergency Coordination and Operations Center, the Police, and the armed forces. A universal toll-free telephone number was reported to be in existence, but it was not functional. The Health Management Information System (HMIS) office at the Ministry of Health did not have information specific to EMS.

#### Health workforce

According to the MOH, four courses addressing health facility-based emergency care were being taught at certain tertiary institutions in the country (Table 2). One additional course (pre-hospital emergency care) was under validation while another was under development.

**Table 2:** EMS courses currently taught at Ugandan tertiary institutions

	Cadre	Course	Award	Duration	Curriculum status
**Pre-hospital**					
Community	Lay first responder	Emergency First Aid Responder-	Certificate	3 days	Under validation
Ambulance	Ambulance Officer	Emergency Medical Technician –Basic (EMT-Basic)	Certificate	5 weeks	Under development
**Health facility**					
Hospital	Nurse	Diploma Emergency Medicine	Diploma	2 years	Running
		Diploma Emergency Nursing	Diploma	2 years	Curriculum under development
		Master of Science Critical Care Nursing	Master’s	2 years	Running
	Clinical Officer	Diploma Emergency Medicine	Diploma	2 years	Running
	Doctor	Master of Medicine Emergency Medicine	MMED	3 years	Running

### Sub-national level findings

#### Governance

EMS delivery was plagued by poor coordination and communication. For instance, of the 11 districts with more than one EMS provider, only 5 (45.5%) coordinated their activities on regular basis. In addition, of the 52 pre-hospital care providers interviewed, 19 (36.5%) reported having dedicated emergency numbers for their ambulances and 25 (48.1%) had designated personnel to handle emergency calls. Twenty-three (44.2%) of the 52 pre-hospital care providers reported use of a central dispatch point for their ambulances. The rest had no specific dispatch mechanism.

#### Medical products

There was widespread lack of the most basic of equipment and medicines (e.g., blood pressure machines, electrocardiogram, glucometer, defibrillator, and forceps) needed to monitor and treat emergency conditions in ambulances and at emergency units (Tables 3, 4 and 5). While triage stations were almost universally available, the capabilities in emergency units to appropriately intervene in life threatening emergencies was limited.

**Table 3:** Availability of equipment and medicines found in ambulances. This is a comparison of ambulances run by varying levels of health care providers including hospitals and stand-alone pre-hospital provider (PHP)

		Level of health facility that manages ambulance			(8) Stand-alone PHP n (%)
	Equipment and Medicines	(5) Referral Hospital n (%)	(20) District Hospital n (%)	(17) Health Centers n (%)	
**Ambulance equipment and emergency medicine available**	Epinephrine (%)	1 (20.0)	9 (45.0)	5 (29.4)	1 (12.5)
	IV fluids (%)	1 (20.0)	12 (60.0)	5 (29.4)	5 (62.5)
	Tranexamic acid (%)	0 (0.0)	1 (5.0)	0 (0.0)	1 (12.5)
	Salbutamol (%)	0 (0.0)	6 (30.0)	4 (23.5)	3 (37.5)
	Pain medication (%)	0 (0.0)	2 (10.0)	2 (11.8)	1 (12.5)
**Airway management equipment**	Suction device (%)	0 (0.0)	5 (25.0)	0 (0.0)	4 (50.0)
	Non-rebreather face mask (%)	0 (0.0)	4 (20.0)	1 (5.9)	4 (50.0)
	Tongue depressor (%)	1 (20.0)	4 (20.0)	1 (5.9)	1 (12.5)
	Nasopharyngeal airway (%)	0 (0.0)	5 (25.0)	1 (5.9)	6 (75.0)
	Oropharyngeal airway (%)	1 (20.0)	3 (15.0)	1 (5.9)	4 (50.0)
**Advanced life support equipment**	Vital signs monitor (%)	0 (0.0)	1 (5.0)	1 (5.9)	0 (0.0)
	ECG (%)	0 (0.0)	0 (0.0)	0 (0.0)	0 (0.0)
	Defibrillator (%)	0 (0.0)	0 (0.0)	0 (0.0)	0 (0.0)
	Intubation set (%)	0 (0.0)	0 (0.0)	0 (0.0)	0 (0.0)

**Table 4:** Availability of equipment and medicines at the health facilities. A comparison of the availability of key equipment and medicines at the different levels of the health care system

	Level of health facility		
	(9) Referral Hospital n (%)	(27) District Hospital n (%)	(68) Health Centers n (%)
**Triage components at the facilities**			
Triage station	9 (100.0)	26 (96.3)	67 (98.5)
Formal triage protocols	8 (88.9)	18 (66.7)	40 (58.8)
Designated triage personnel	8 (88.9)	20 (74.1)	50 (73.5)
Time targets for certain triage designation	4 (44.4)	10 (37.0)	8 (11.8)
Compliance tracking for triage time target	3 (33.3)	5 (18.5)	2 (2.9)
**Equipment and supplies for managing circulation in the emergency unit**			
Different size cannulas	9 (100.0)	27 (100.0)	60 (88.2)
Crystalloids	8 (88.9)	27 (100.0)	64 (94.1)
Dextrose	9 (100.0)	27 (100.0)	63 (92.6)
Central venous catheters	0 (0.0)	2 (7.4)	1 (1.5)
Fluid administration set	9 (100.0)	27 (100.0)	66 (97.1)
Blood administration set	7 (77.8)	19 (70.4)	13 (19.1)
**Equipment for managing breathing in the emergency unit**			
Different size cannulas	6 (66.7)	20 (74.1)	28 (41.2)

**Table 5:** Functional status of available equipment in the health facility. A comparison of the number of functional equipment at the different levels of the health care system

	Level of health facility		
	(9) Referral Hospital a/b (%)	(27) District Hospital a/b (%)	(68) Health Centers a/b (%)
**Equipment for managing the airway in the emergency unit**			
McGill forceps (%)	0 (NaN)	7/7 (100.0)	1/1 (100.0)
Suction apparatus (%)	6/7 (85.7)	14/15 (93.3)	16/20 (80.0)
Laryngoscope (%)	3/3 (100.0)	8/9 (88.9)	9/9 (100.0)
Nasopharyngeal airway (%)	1/1 (100.0)	5/5 (100.0)	2/2 (100.0)
Oropharyngeal (adult) (%)	4/5 (80.0)	10/11 (90.9)	10/11 (90.9)
Endotracheal tube ETT (%)	2/3 (66.7)	8/8 (100.0)	7/8 (87.5)
Tracheostomy set (%)	3/3 (100.0)	5/5 (100.0)	0/0 (NaN)
**Equipment for managing the airway in the emergency unit**			
Oxygen cylinder (%)	7/9 (77.8)	15/16 (93.8)	16/20 (80.0)
Nasal prongs (%)	8/8 (100.0)	16/19 (84.2)	15/19 (78.9)
Chest tube and underwater seal drainage (%)	2/2 (100.0)	4/4 (100.0)	0/0 (NaN)
Mechanical ventilator (%)	0 (NaN)	3/3 (100.0)	1/3 (33.3)
**Equipment and supplies for managing circulation in the emergency unit**			
ECG machine (%)	2/3 (66.7)	9/9 (100.0)	2/3 (66.7)
Defibrillator (%)	1/1 (100.0)	4/4 (100.0)	1/1 (100.0)
**Equipment and supplies for immobilisation and splinting**			
Cervical collar (soft/hard collar) (%)	2/2 (100.0)	7/7 (100.0)	5/5 (100.0)
Spine board (%)	1/1 (100.0)	1/1 (100.0)	1/1 (100.0)

a = Number of functional equipment available, b = Total number of equipment available NaN = Equipment not available at this level of health facilities

Seventy-nine (71.2%) of the 111 sampled emergency units reported use of clinical protocols. However, except for a few wall charts dealing with disease-specific conditions, there was little evidence of protocol use. Private-owned health facilities and ambulances were relatively better equipped and stocked than government-owned ones.

#### Health workforce

The Police, which was responsible for most (69%) casualty transfers, had no trained medical personnel on board. They also used pick-up trucks with no provision for patient space beyond the bare floor of the truck. The rest of the providers had ambulances. Nine of the sixteen (56.3%) non-Police pre-hospital providers with ambulances assessed had a nurse on board, with some having an additional health worker (e.g., emergency medical technician or doctor) when handling emergencies. Ninety-four percent (15/16) of the non-Police pre-hospital providers had ambulance workers on a fixed salary, in addition to a variety of other remuneration mechanisms (e.g., allowances and pay-per-rescue).

Only 27% (30/111) of the sampled health facilities had permanent (non-rotating) staff in their emergency unit. This was evident even at the regional referral hospital level, where only three of the seven regional referral hospitals had permanent emergency room staff. Furthermore, 91% (101/111) of the emergency personnel in the sampled health facilities (regardless of the level of care), had received no specific training in the management of emergencies.

#### Service delivery

Nearly one in three (28.9%) of the sampled districts had more than one pre-hospital emergency services provider. The same proportion had a medical director, with 66% of the pre-hospital service providers having no medical director. Fifty (44.6%) of the 112 health facilities and police stations assessed for ambulance services had an ambulance (42 standard ambulances^1^ and 8 improvised ambulances).

Government (particularly the police) as well as private for-profit and not-for-profit agencies provided pre-hospital care services. Thirty-three (63.5%) of the 52 pre-hospital EMS providers were government-funded, with for-profit and not-for-profit agencies funding the remainder. Of the 52 pre-hospital providers, 16 (30.8%) reported having ambulance services with vehicles while the remaining 36 who were the Uganda Police had no ambulances but improvised means of transportation (Police patrol trucks) in emergency situations. The median cost for a long ambulance run was 114 US dollars (400,000 Uganda shillings) with a range between 9 US dollars (30,000 Uganda shillings) and 943 US dollars (3,300,000 Uganda shillings). This excludes government-owned pre-hospital care providers (especially the Police) who generally provide free transportation to health facilities. Forty-two (84%) of the 50 pre-hospital care providers that had ambulance services were attached to a health facility. Only 8 (16%) of these providers were stand-alone (i.e., not attached to a health facility).

While it was expected that lower levels of the healthcare system (i.e., HC III and HC IV) might be closed for the night, we found 18.4% of hospitals, including regional referral hospitals, where emergency services were not available 24 h a day. Forty-two (37.8%) of the 111 sampled health facilities did not have laboratory support for part of the day. Moreover, there was little capacity to manage extra-ordinary events such as mass causality events at all levels of the healthcare system.

#### Information

There was wide variation in the formats and types of data collected on EMS within districts. Most information was neither shared with relevant agencies and offices, nor was there much indication that it influenced planning. Even operations-specific information such as the fact that critically ill patients were being transferred to a certain health facility was not always shared. For instance, 26 (50%) of the 52 EMS providers interviewed reported that they never notified health facilities prior to transferring emergencies there. Only 13 (34%) of the 38 sampled districts used their EMS data for district-level system planning. Data for planning EMS came from health facility records, police records, mortuary records, and community sources. Thirty (57.7%) of the 52 pre-hospital care providers reported sharing their information with authorities at the district level while 17 (32.7%) shared their data with the MOH. Other stakeholders with whom data were shared included District Police Commanders, the National Road Safety Council, and the media.

## Discussion

Our study found an unstructured emergency medical services system hobbled by lack of national policy, guidelines, and standards; funding; medical products, and coordination. Ambulances and emergency areas in health facilities lacked the most basic of equipment and medicines both to monitor and to treat emergency medical conditions. This severe lack of equipment and medicines was observed at all levels of the health system regardless of level or ownership of the facility or the ambulance, although private health facilities and ambulances were relatively better equipped and stocked than government ones. The limited availability and functionality of medical equipment for responding to emergency medical conditions meant patients were getting very limited care in the pre-hospital phase, and then being transported to health facilities that were only marginally better equipped to manage their acute events.

Service delivery was plagued by poor coordination and communication. At least 50% of the EMS providers interviewed reported that they never notified health facilities prior to transferring emergencies there. That hospitals, including regional referral hospitals did not have EMS available 24 h a day and police patrol vehicles (pick-up trucks) were the commonest (36 of 52 providers) mode of transporting patients in need of emergency care is likely a reflection of the resource challenges across the health care system. Because the study had defined an ambulance as one providing both emergency transportation and care while in the pre-hospital space, it meant that the majority of pre-hospital providers did not have ambulances, but they were providers of emergency transportation. Moreover, at every level, there was evidence of insufficient financing for EMS.

Despite these challenges, the MOH has shown commitment to improving the state of EMS in the country. This was evident through the recent establishment of a department for emergency medical services in the MOH and the setting up of a special taskforce to spearhead the development of national EMS documents including policies, guidelines, and standards.

Our findings corroborate those from other studies using similar methodology which found lack of leadership, legislation and funding as key barriers to the development of EMS in developing countries [16]. This was a national survey and therefore the findings could be generalized to the whole of Uganda. The findings could also be generalized to other low- and middle-income countries within Africa that have no EMS systems [1] and can therefore be used to guide efforts aimed at improving EMS systems within these settings.

A limitation of this study is the potential for measurement error from reliance on self-reports for some of the outcomes (e.g., data use for planning), which could have resulted into social desirability bias. However, majority of the key outcomes (availability and functionality of medical products) in the study were measured through direct observation. Although the findings of the study are geared towards policy makers in Uganda especially the Ministry of Health, any persons and entities can use them for advocacy or further research.

## Conclusion

While it is not in doubt that Uganda has a multi-tiered system of health facilities to which patients can go for medical care, our findings for the pre-hospital care component beg the question, ‘Does Uganda have an EMS?’ This study was conducted at a time when there was no EMS policy, no standards, and very poor coordination at national and sub-national levels. However, there was a process underway to develop policies and guidelines for the establishment of the EMS. From our findings, it therefore seems prudent to conclude that there was in fact no EMS, but a number of important components were in place which could be restructured as a starting point for the establishment of the system. This conclusion would be consistent with a 2017 study of EMS across Africa, which found that Uganda had no EMS system [2].

## Data Availability

The datasets used and/or analysed during the current study are available on reasonable request. The corresponding author [AN] can work with interested researchers to secure approval from the relevant authorities (Ministry of Health) to reuse the dataset for research.

## Abbreviations

ECSSA: Emergency Care Systems Assessment
EMS: Emergency Medical Services
EMT: Emergency Medical Technician
HC: Health Center
HMIS: Health Management Information System
MoH: Ministry of Health
NGO: Non-Governmental Organization
ODK: Open Data Kit
PNFP: Private Not-For-Profit
RRH: Regional Referral Hospital
WHO: World Health Organization

## Footnotes

1 A standard ambulance was defined as a motor vehicle licensed under the *Uganda Traffic & Road Safety Act* and marked as such, designed or adapted for the treatment and conveyance of patients in an emergency.
